# Sexually transmitted infections and risk of hypertensive disorders of pregnancy

**DOI:** 10.1038/s41598-022-17989-0

**Published:** 2022-08-16

**Authors:** Brandie DePaoli Taylor, Ashley V. Hill, Maria J. Perez-Patron, Catherine L. Haggerty, Enrique F. Schisterman, Ashley I. Naimi, Akaninyene Noah, Camillia R. Comeaux

**Affiliations:** 1 Division of Basic Science and Translational Research, Department of Obstetrics and Gynecology, University of Texas Medical Branch, Galveston, TX USA; 2 Department of Preventive Medicine and Population Health, University of Texas Medical Branch-Galveston, Galveston, TX USA; 3 Department of Epidemiology, School of Public Health, University of Pittsburgh, Pittsburgh, PA USA; 4 Department of Epidemiology and Biostatistics, School of Public Health, Texas A&M University, College Station, TX USA; 5 Department of Biostatistics, Epidemiology and Informatics, Perelman School of Medicine, University of Pennsylvania, Philadelphia, PA USA; 6 Department of Epidemiology, Rollins School of Public Health, Emory University, Atlanta, GA USA

**Keywords:** Diseases, Medical research, Risk factors

## Abstract

Hypertensive disorders of pregnancy (HDP) result in maternal morbidity and mortality but are rarely examined in perinatal studies of sexually transmitted infections. We examined associations between common sexually transmitted infections and HDP among 38,026 singleton pregnancies. Log-binomial regression calculated relative risk (RRs) and 95% confidence intervals (CIs) for associations with gestational hypertension, preeclampsia with severe features, mild preeclampsia, and superimposed preeclampsia. All models were adjusted for insurance type, maternal age, race/ethnicity, and education. Additional adjustments resulted in similar effect estimates. Chlamydia was associated with preeclampsia with severe features (RR_adj_. 1.4, 95% CI 1.1, 1.9). Effect estimates differed when we examined first prenatal visit diagnosis only (RR_adj_. 1.3, 95% CI 0.9, 1.9) and persistent or recurrent infection (RR_adj_. 2.0, 95% CI 1.1, 3.4). For chlamydia (RR_adj_. 2.0, 95% CI 1.3, 2.9) and gonorrhea (RR_adj_. 3.0, 95% CI 1.1, 12.2), women without a documented treatment were more likely to have preeclampsia with severe features. Among a diverse perinatal population, sexually transmitted infections may be associated with preeclampsia with severe features. With the striking increasing rates of sexually transmitted infections, there is a need to revisit the burden in pregnant women and determine if there is a link between infections and hypertensive disorders of pregnancy.

## Introduction

*Chlamydia trachomatis*, syphilis, and *Neisseria gonorrhoeae* are common sexually transmitted infections (STIs) that can lead to adverse pregnancy outcomes^1^. The Centers for Disease Control (CDC) recommends universal screening for syphilis in the first trimester and screening for chlamydia and gonorrhea for women < 25 years of age or at high risk. However, these pathogens have been increasing annually since 2014^2^ with glaring racial/ethnic disparities. To optimize screening and prevention tactics, research needs to determine the burden of non-viral STIs on specific adverse birth outcomes, particularly in diverse populations^3^. Meta-analyses are limited but studies examining associations between non-viral STIs and adverse pregnancy outcomes are inconsistent and many suffer from confounding, rely on International Classification of Diseases (ICD)-9 codes or birth certificate data^4,5^ and/or have suboptimal screening in the source population (e.g. < 60% are screened)^1,6,7^. Many studies are greater than 10 years old and focused on preterm birth, low birth weight, and stillbirth, excluding hypertensive disorders of pregnancy.

Hypertensive disorders of pregnancy (HDP) are characterized by elevated blood pressure during pregnancy. Recent data reports rates as high as 16% in Black women, 13.4% in White women, and 10.6% in Hispanic women^8^. Immune dysfunction is one specific pathway leading to inadequate invasion of trophoblasts, inadequate placentation, endothelial dysfunction and the development of hypertension during pregnancy^9^. It is accepted that maternal factors influence the response to placental dysfunction resulting in clinical symptoms, but maternal genital infections known to trigger inflammatory pathways are not often examined within the context of HDP.

Innate and T-cell immunity plays a role in *C. trachomatis* and *N. gonorrhoeae* induced reproductive tissue damage^10^. *Chlamydia muridarum* accelerates atherosclerosis and production of systemic inflammation in murine models^11^. These pathways overlap with hypertension and preeclampsia pathogenesis^9^. An association between serological evidence^12,13^ and nucleic acid amplification test diagnosis^14,15^ of *Chlamydia trachomatis* and preeclampsia have been reported. These studies did not examine persistent or recurrent infection, underlying chronic hypertension, or treatment. One study examined preeclampsia with severe features, but this small study relied on serology for diagnosis^13^. Far fewer studies have examined gonorrhea and syphilis and HDP. Our study aims to expand on the current literature and determine if STIs are associated with severe subtypes of HDP, while considering persistent or recurrent infection and treatment.

## Results

### Population characteristics

Overall, majority of those in our study were Hispanic (57.6%), foreign born (51.0%), between the ages of 30–39 (45.7%), married (70.3%), where on Medicaid/Children’s Health Insurance Program (CHIP) (62.6%), had a high school education or less (51.3%), had one or more prior pregnancies (61.7%), and received prenatal care in the first trimester (59.9%). Both smoking (0.9%) and drug use (0.44%) were low in our population. Women who were ≥ 25 years (PR 0.2, 95% CI 0.1, 0.3), had one or more prior pregnancies (PR 0.7, 95% CI 0.6, 0.9), and self-identified as White (PR 0.7, 95% CI 0.6, 0.8) or Asian (PR 0.2, 95% CI 0.1, 0.3) were less likely to have a STI (Table 1). Women who had less than high school education (PR 2.9, 95% CI 2.6, 3.2), were single (PR 3.2, 95% CI 2.9, 3.6), and on Medicaid/(CHIP) (PR 5.7, 95% CI 4.8, 6.8) were more likely to have a STI. Prenatal tobacco use (PR 1.9, 95% CI 1.3, 2.8), drug use (PR 1.4, 95% CI 1.2, 1.7) and first prenatal visit in the second (PR 1.9, 95% CI 1.7, 2.1) and third trimesters (PR 2.1, 95% CI 1.8, 2.5) were associated with a STI diagnosis.

**Table 1:** Maternal demographic and clinical characteristics by non-viral sexually transmitted infection (STI) status.

Variable	Missing n (%)	STI^+^ n (%)	STI^–^ n (%)	Prevalence ratio (95% CI)
**Demographic characteristics**				
Age	NA			
< 25		1083 (10.0)	9738 (90.0)	Ref
≥ 25		731 (2.7)	26,474 (97.3)	0.2 (0.1–0.3)
Ethnicity	31 (< 1%)			
Non-Hispanic		574 (3.6)	15,326 (96.4)	Ref
Hispanic		1239 (5.6)	20,856 (94.4)	1.6 (1.4–1.7)
Race/ethnicity	31 (< 1%)			
White vs. non-white		1303 (4.4)	28,366 (95.6)	0.7 (0.6–0.8)
Black vs. non-black		454 (7.7)	5417 (92.3)	1.8 (1.7–2.0)
Asian vs. non-Asian		23 (1.2)	1845 (98.8)	0.2 (0.1–0.3)
Native Hawaiian/PI		2 (5.4)	35 (94.6)	–
American Indian/NA		1 (4.6)	21 (95.5)	–
Other race vs. non-other		35 (4.8)	696 (95.2)	1.0 (0.7–1.4)
Education	3561 (9.4%)			
HS or greater		420 (2.5)	16,350 (97.5)	Ref
< HS education		1220 (6.9)	16,475 (93.1)	2.9 (2.6–3.2)
Marital status	690 (1.8%)			
Married		782 (3.0)	25,482 (97.0)	Ref
Single		1000 (9.0)	10,072 (91.0)	3.2 (2.9–3.6)
Method of payment	601 (1.6%)			
Private		141 (1.2)	11,752 (98.8)	Ref
Medicaid/CHIP		1503 (6.4)	21,925 (93.6)	5.7 (4.8–6.8)
No-insurance/other		137 (6.5)	1967 (93.5)	5.8 (4.6–7.3)
**Behavioral characteristics and health**				
Prenatal alcohol use	14 (< 1%)			
No		1801 (4.8)	35,860 (95.2)	Ref
Yes		11 (3.1)	340 (96.9)	0.7 (0.4–1.2)
Prenatal smoking	12 (< 1%)			
No		1784 (4.7)	35,903 (95.3)	ref
Yes		29 (8.9)	298 (91.1)	1.9 (1.3–2.8)
Prenatal drug use	27 (< 1%)			
No		1653 (4.7)	33,858 (95.4)	Ref
Yes		159 (6.4)	2329 (93.6)	1.4 (1.2–1.7)
BMI kg/m^2^	304 (< 1%)			
< 24.9		298 (5.1)	5541 (94.9)	Ref
25–29.9		567 (4.9)	10,887 (95.1)	1.0 (0.8–1.1)
30+		935 (4.6)	19,494 (95.4)	0.9 (0.8–1.0)
Chronic hypertension	218 (< 1%)			
No		1714 (4.8)	34,122 (95.2)	Ref
Yes		86 (4.4)	1886 (95.6)	0.9 (0.7–1.1)
Cardiovascular disease	218 (< 1%)			
No		1785 (4.7)	36,073 (95.3)	Ref
Yes		19 (4.4)	414 (95.6)	0.9 (0.6–1.5)
Diabetes	218 (< 1%)			
No		1781 (4.8)	35,597 (95.2)	Ref
Yes		19 (4.4)	411 (95.6)	0.7 (0.5–1.1)
**Pregnancy history and current pregnancy**				
Prior pregnancy	9 (< 1%)			
Nulliparous		626 (5.8)	10,159 (94.2)	Ref
1+ prior pregnancy		1187 (4.4)	26,045 (95.6)	0.7 (0.6–0.9)
Prior spontaneous abortion	17 (< 1%)			
No		1363 (5.1)	25,485 (94.9)	Ref
Yes		451 (4.0)	10,710 (96.0)	0.8 (0.7–0.9)
Prior preterm birth	17 (< 1%)			
No		1672 (4.8)	32,973 (95.2)	Ref
Yes		142 (4.2)	3222 (95.8)	0.9 (0.7–1.0)
GA at 1st prenatal visit	NA			
≤ 12 weeks		910 (3.7)	23,945 (96.3)	Ref
13–26 weeks		727 (6.7)	10,049 (93.3)	1.9 (1.7–2.1)
27+ weeks		177 (7.4)	2218 (92.6)	2.1 (1.8–2.5)
Infant sex	37 (< 1%)			
Female		850 (4.6)	17,695 (95.4)	Ref
Male		962 (4.9)	18,482 (95.1)	1.1 (1.0–1.2)

Log-binomial logistic regression was used to calculate point prevalence ratios and 95% confidence intervals.

*PI* Pacific Islander, *HS* High School, *BMI* body mass index, *CHIP* Children’s Health Insurance Program, *GA* gestational age.

### Sexually transmitted infections and hypertensive disorders of pregnancy

A total of 1521 women (4.0%) were diagnosed with *C. trachomatis* at the first prenatal visit, 150 (0.39%) were diagnosed with *N. gonorrhoeae*, and 268 (0.70%) were diagnosed with syphilis. Chlamydia (RR_adj_. 1.2, 95% CI 1.1, 1.5) and syphilis (RR_adj_. 1.5, 95% CI 1.1, 2.3) were associated with gestational hypertension (Table 2). Chlamydia was associated with preeclampsia with severe features (RR_adj_. 1.4, 95% CI 1.1, 1.9) and superimposed preeclampsia (RR_adj_. 1.6, 95% CI 1.1, 2.4). Gonorrhea was not associated with HDP, although a similar percentage as chlamydia (3.3% vs. 3.2%) developed preeclampsia with severe features (RR_adj_. 1.4, 95% CI 0.5, 3.0). Rates of HDP were similar among chlamydia positive women who received testing in the first trimester (20.9% for chlamydia positive vs. 16.4% for chlamydia negative) and testing in the second or third trimesters (19.3% vs. 17.0%). For example, effect estimates for the association between chlamydia and preeclampsia with severe features were similar with confidence interval overlap for first trimester testing (3.1%: RR_adj_. 1.5, 95% CI 0.9, 2.3) and second/third trimester testing (3.2% RR_adj_. 1.8, 95% CI 1.1, 2.7).

**Table 2:** Sexually transmitted infections (STI) and hypertensive disorders of pregnancy.

Sexually transmitted infection	Gestational Hypertension n (%) *RR_adj._ (95% CI)	Mild PE n (%) *RR_adj._ (95% CI)	+ PE with severe features n (%) *RR_adj._ (95% CI)	Superimposed PE n (%) *RR_adj._ (95% CI)
No STI, n = 36,698	n = 2934 (8.0%)	n = 1388 (3.6%)	n = 688 (1.9%)	n = 608 (1.8%)
**Chlamydia, n = 1521**	n = 147 (11.0%)	n = 69 (5.3%)	n = 48 (3.2%)	n = 29 (2.1%)
Crude	1.2 (1.0–1.4)	1.2 (0.9–1.5)	1.7 (1.2–2.2)	1.2 (0.9–1.7)
Model 1	1.2 (1.0–1.5)	1.1 (0.8–1.4)	1.4 (1.1–1.9)	1.5 (1.1–2.1)
Model 2	1.2 (1.1–1.5)	1.1 (0.8–1.4)	1.4 (1.1–1.9)	1.6 (1.1–2.1)
**Gonorrhea, n = 150**	n = 15 (9.7%)	n = 8 (5.4%)	n = 5 (3.3%)	n = 3 (2.2%)
Crude	0.9 (0.6–1.7)	1.0 (0.5–2.3)	1.7 (0.7–4.1)	++
Model 1	1.0 (0.5–1.7)	1.0 (0.5–1.8)	1.5 (0.6–3.5)	
Model 2	0.8 (0.4–1.5)	1.1 (0.5–2.6)	1.4 (0.5–3.0)	
**Syphilis, n = 268**	n = 33 (14.0%)	n = 11 (4.9%)	n = 6 (2.2%)	n = 7 (2.8%)
Crude	1.5 (1.1–2.1)	1.1 (0.6–2.0)	1.1 (0.6–2.5)	1.6 (0.8–3.4)
Model 1	1.6 (1.1–2.2)	1.1 (0.6–1.9)	1.0 (0.5–2.3)	1.5 (0.9–2.6)
Model 2	1.5 (1.1–2.3)	1.1 (0.7–2.3)	1.0 (0.4–2.2)	1.4 (0.8–2.5)

*Model 1: Adjusted for insurance type, maternal age, race/ethnicity, and education.

*Model 2: Fully adjusted model included all variables in model 1 plus smoking, substance use and co-infection with other STIs.

^+^Preeclampsia with severe features as defined by American College of Obstetricians and Gynecologists guidelines.

### Influence of treatment for sexually transmitted infections on hypertensive disorders of pregnancy

Among women with chlamydia, 58.0% had evidence of CDC recommended treatment in the electronic health record. Results were similar for gonorrhea (52%) and syphilis (43.7%). Not having a documented CDC recommended treatment for chlamydia was associated with preeclampsia with severe features (RR_adj_. 2.0, 95% CI 1.3, 2.9) and superimposed preeclampsia (RR_adj_. 2.2, 95% CI 1.3, 3.6). We identified a similar trend with gonorrhea for preeclampsia with severe features (RR_adj_. 3.0, 95% CI 1.1, 12.2), although the small sample size for this analysis resulted in an imprecise estimate. These trends were not observed for syphilis.

### Persistent or recurrent chlamydia and hypertensive disorders of pregnancy

Women with a diagnosis of chlamydia at the first prenatal visit only and no subsequent diagnosis in the third trimester, did not have increased risk of HDP, expect for superimposed preeclampsia (RR_adj_. 1.7, 95% CI 1.1, 2.6). Persistent or recurrent chlamydia infection was associated with preeclampsia with severe features (RR_adj_. 2.0, 95% CI 1.1, 3.4) (Table 3). Sample sizes were not large enough to examine gonorrhea and syphilis.

**Table 3:** *Chlamydia trachomatis* and risk of hypertensive disorders of pregnancy by single diagnosis and persistent or recurrent infection.

Chlamydia	Gestational Hypertension n (%) *RR_adj._ (95% CI)	Mild PE n (%) *RR_adj._ (95% CI)	+ PE with severe features n (%) *RR_adj._ (95% CI)	Superimposed PE n (%) *RR_adj._ (95% CI)
No chlamydia, n = 36,698	n = 2934 (8.0%)	n = 1388 (3.6%)	n = 688 (1.9%)	n = 608 (1.8%)
**Single diagnosis, n = 1333**	n = 128 (9.6%)	n = 60 (4.5%)	n = 38 (2.9%)	n = 27 (2.0%)
Crude	1.2 (1.0–1.4)	1.2 (0.9–1.6)	1.5 (1.1–2.1)	1.2 (0.9–1.8)
Model 1	1.2 (1.0–1.5)	1.0 (0.8–1.4)	1.4 (0.9–1.9)	1.8 (1.2–2.6)
Model 2	1.2 (1.0–1.5)	1.0 (0.8–1.4)	1.3 (0.9–1.9)	1.7 (1.1–2.6)
**Persistent or recurrent infection, n = 192**	n = 19 (9.8%)	n = 9 (4.7%)	n = 10 (5.2%)	n = 2 (1.0%)
Crude	1.2 (0.8–1.9)	1.3 (0.7–2.4)	2.7 (1.5–5.0)	++
Model 1	1.3 (0.8–2.0)	1.1 (0.6–2.1)	2.0 (1.1–3.8)	
Model 2	1.2 (0.8–2.0)	1.1 (0.6–0.5)	2.0 (1.1–3.4)	

Single diagnosis refers to women diagnosed at the first prenatal visit only, persistent or recurrent infection refers to an additional diagnosis recorded after repeat testing.

*Model 1: Adjusted for insurance type, maternal age, race/ethnicity, and education.

*Model 2: Fully adjusted model included all variables in model 1 and smoking, substance use and co-infection with other STIs.

^+^Preeclampsia with severe features as defined by American College of Obstetricians and Gynecologists guidelines.

### Sensitivity analyses

We estimated E-values (Supplementary Tables 1, 2), minimal strength of association with exposure and outcome the unmeasured confounder would need to have to bias results after considering adjustment for all included covariates. For example, the association between chlamydia and gestational hypertension, the E-value (1.7) for the point estimate and confidence interval (1.4) were close to one, thus a small or moderate degree of unmeasured confounding could explain these results. Other E-values ranged from 2.6 (1.4 for confidence interval) for the association with chlamydia and preeclampsia with severe features to 3.4 (1.4 for the confidence interval) for the association between a second chlamydia diagnosis and preeclampsia with severe features.

## Discussion

Much of the data on the association between non-viral STIs and specific adverse pregnancy outcomes is based on outdated studies^3^, most of which lack diversity and do not consider treatment or persistence/recurrence of infections. We found that *Chlamydia trachomatis* is associated with preeclampsia with severe features and superimposed preeclampsia. We observed that associations with severe disease were exacerbated in women with persistent or recurrent infection and among those without a documented CDC recommended treatment. Gonorrhea positive women without a documented treatment also had increased risk of preeclampsia with severe features. Lastly, we observed an association between syphilis and gestational hypertension.

Our study addressed limitations of prior research in a modern diverse cohort. A nested-case control study of 628 women from the Collaborative Perinatal Project, found a significant association between IgG antibody titers for chlamydia and preeclampsia^12^. In a nested case–control study of 845 women from the Danish National Birth Cohort, there were trends towards associations between serological evidence of chlamydia and preeclampsia, preeclampsia with severe features, and preterm preeclampsia^13^. Results were not statistically significant but chlamydia was uncommon in that study. In a smaller subset of our study population, chlamydia detected by Nucleic Acid Amplification Test (NAAT) was associated with term preeclampsia among women < 25 years of age^14^. The study did not examine other HDP, did not consider superimposed preeclampsia, and did not examine severity of disease, which is a significant contributor to maternal morbidity and mortality. None of these studies examined additional chlamydia diagnoses throughout pregnancy nor evidence of recommended treatments.

To our knowledge, very few studies have examined the association between gonorrhea and HDP. In a meta-analysis of 33 studies^6^, there were moderate associations between gonorrhea and preterm birth, pPROM, and perinatal mortality, but most studies did not adjust for confounders. One retrospective cohort utilizing vital records and ICD-9 codes from California based hospitals, found no association between syphilis and HDP^16^. Birth certificate data and ICD-9 codes have reduced reliability for prenatal visit data and maternal complications^17^.

Suboptimal screening for sexually transmitted infections (~ 60%), delays in treatment (55%), and persistent or recurrent infection (9–14%) are common during pregnancy, particularly in socially disadvantaged populations^3,18^. A recent study among 810 women attending clinics in Alabama who were treated with azithromycin for chlamydia had a recurrence rate of 9% and persistence rate of 14%^18^. In that study, HDP were not examined as outcomes. In our study, ~ 13% had a second diagnosis, which may be an underestimation. Thus, efforts are needed to ensure that women are receiving prompt screening and treatment adherence. Additional research is needed to determine if expedited partner therapy or other public health interventions may reduce STI recurrence or persistence and subsequent adverse pregnancy outcomes in these populations.

Immune adaptation is closely involved in pregnancy success as the decidua contains macrophages, natural killer cells, and regulatory T-cells that are in intimate contact with trophoblasts^19^. In preeclampsia, immune dysregulation is one pathway which may result in inadequate invasion of trophoblast and subsequent placental hypoxia, oxidative stress, systemic inflammation, and endothelial dysfunction^9^. However, preeclampsia is a syndrome suggested to have multiple subtypes, which may have different pathologies^20^. We did not identify associations across all subtypes of preeclampsia. For example, chlamydia was associated with preeclampsia with severe features but not mild preeclampsia. While we also found an association between chlamydia and superimposed preeclampsia, ~ 17% of individuals with superimposed preeclampsia developed severe features. This could suggest that *C. trachomatis* results in pathophysiological changes that increase the probability of developing severe disease among those with preeclampsia. While subtypes of preeclampsia are not well characterized, Leavey et al. utilizing placental gene expression identified an immunological cluster that correlated with severe maternal outcomes^21^. It has been suggested that the maternal microbiota is important for ensuring maternal immune regulation and ultimately receptivity^19^. Bacterial STIs including *C. trachomatis* are known to interact with the vaginal microbiome^22^. It is possible that infections could disrupt the maternal immune milieu needed for proper implantation and placentation.

The mechanisms linking either chlamydia, syphilis, or gonorrhea to HDP have not been identified. However, *C. trachomatis* and *N. gonorrhoeae* infection results in changes to immune pathways that are relevant in preeclampsia pathogenesis (innate immunity, T-cells)^10^. In addition, chlamydia is associated with increased inflammatory marker expression on trophoblasts^23^, and *C. muridarum* accelerates atherosclerosis and production of systemic inflammation in murine models^11^. Both chlamydia and syphilis are associated with mechanisms involved in cardiovascular disease^11,24^, which overlaps with pathways involved in preeclampsia. Further studies are needed to determine the mechanisms that may explain our observed associations. Additional observational studies are needed to determine if results can be replicated in different populations, particularly to determine if the association between chlamydia and preeclampsia with severe features is consistent. Experimental and animal models will be required to better understand mechanism, as one limitation of perinatal cohorts is an inability to determine the effect of exposures on early pregnancy processes, such as decidualization, implantation, and placentation.

Our study had several strengths, including the utilization of electronic health records from a large diverse population of pregnant women. Approximately 90–97% of our cohort had diagnostic tests for chlamydia, gonorrhea, and syphilis. This is compared to other retrospective studies where < 25% of birth records had STI diagnostic tests completed^7^ and 60% had STI diagnostic tests completed based on laboratory data^1^. Thus, while the use of existing data is a limitation of retrospective cohorts, Peribank has a robust database that minimizes bias. However, our study design can result in selection bias due to exclusion of individuals based on availability of data. Exclusion due to a missing STI test could reasonably be associated with exposure as receiving a test is dependent on age and risk of STI. Because those excluded were slightly more likely to be Black and have no insurance, they reasonably may have a higher risk of exposure. We can also assume that these women without a diagnostic test potentially could have a higher risk of adverse outcomes. If the above scenario is the true then it is possible that cases with exposure could have been excluded, which would bias the results towards the null.

Other limitations are possible with retrospective evaluation of administrative databases. We did not have available data on other important variables. We cannot distinguish whether persistence or recurrence or timing of infection was associated with our outcomes. However, given the pathogenesis of preeclampsia is likely to begin very early in pregnancy^25^, chronic inflammation due to persistent or recurrent infection is more plausible than a new infection later in pregnancy. While electronic health records with consistent quality checks allowed us improved phenotyping of HDP, it is an administrative database and misclassification is possible. We did have a low prevalence of gonorrhea (< 1%) but this is consistent with national data among Hispanic women, whom make up the largest portion of our cohort^26^. A high proportion of STI positive individuals did not have a documented treatment in our study. Thus, our results could reflect a delay in treatment, which was not recorded, rather than no treatment at all. In similar studies, ~ 30% of records did not have documented treatments for perinatal STIs^18,27^. Goggins et al.^27^, examined treatment delays reporting that over 50% of patients experienced delayed STI treatment during pregnancy, with an overall range of 0–221 days. These delays were primarily due to lack of clinician recognition of positive results and difficulty contacting patients. The use of secondary data also precluded the inclusion of data on discrimination or other social factors, which might be important unmeasured confounders. As our E-values indicated, it is possible that moderate unmeasured confounding could bias our findings, especially for the association between chlamydia and gestational hypertension. Lastly, we recognized that multiple comparisons increase type I error, but correction of p-values can increase type II error^28,29^. We followed the recommendations of Rothman and others^28,29^ to report effect estimates and confidence intervals rather than p-values, interpreting our results with consideration of potential bias.

Data on the impact of non-viral STIs on pregnancy health are inconsistent and hypertensive disorders of pregnancy are rarely considered within the spectrum of potential outcomes. More recent literature is beginning to find associations between STIs and HDP, supporting additional research in this area. Increasing rates of chlamydia, gonorrhea and syphilis coupled with significant racial/ethnic disparities warrants renewed focus on genital tract infections during pregnancy. In populations where majority of women come from socially disadvantaged backgrounds, our results add to the current literature and suggest that persistent or recurrent genital infections may be associated with preeclampsia with severe features and superimposed preeclampsia.

## Methods

We obtained data from Peribank, a database and biorepository that recruits women from two hospitals within Baylor College of Medicine, a tertiary-care referral center, as well as the Texas Children’s Hospital Pavilion for Women, a private hospital, in Houston, TX^30^. All women are approached at the time of admissions to labor and delivery by trained study personnel and are eligible for enrollment if they meet the inclusion criteria: ≥ 18 years of age or ≥ 16 if emancipated and able to understand and sign the consent form. There are no exclusion criteria. PeriBank has shown previously that they are able to enroll 85–93% of all gravidae presenting to labor and delivery. Given that the hospitals serve a large portion of Hispanic pregnant individuals, this is reflected in the demographic characteristics of Peribank. However, maternal characteristics are comparable to births in the region (Supplementary Table 3). Clinical metadata from electronic medical records and direct questioning is used to create the database by standardized protocols developed by a working group that consisted of board-certified physicians in obstetrics and gynecology, maternal fetal medicine, and other relevant disciplines. For quality control, a subset of charts included in this study were audited by a maternal–fetal medicine physician scientist. All women provided informed consent. Peribank protocols were approved by the Baylor College of Medicine Institutional Review Board and all methods were performed following guidelines and regulations. The Peribank Governance Board provided permission to access the data, which was anonymized prior to use. The current study protocol was reviewed by the University of Texas Medical Branch Institutional Review Board and determined to be non-human subjects research.

**Figure 1:**
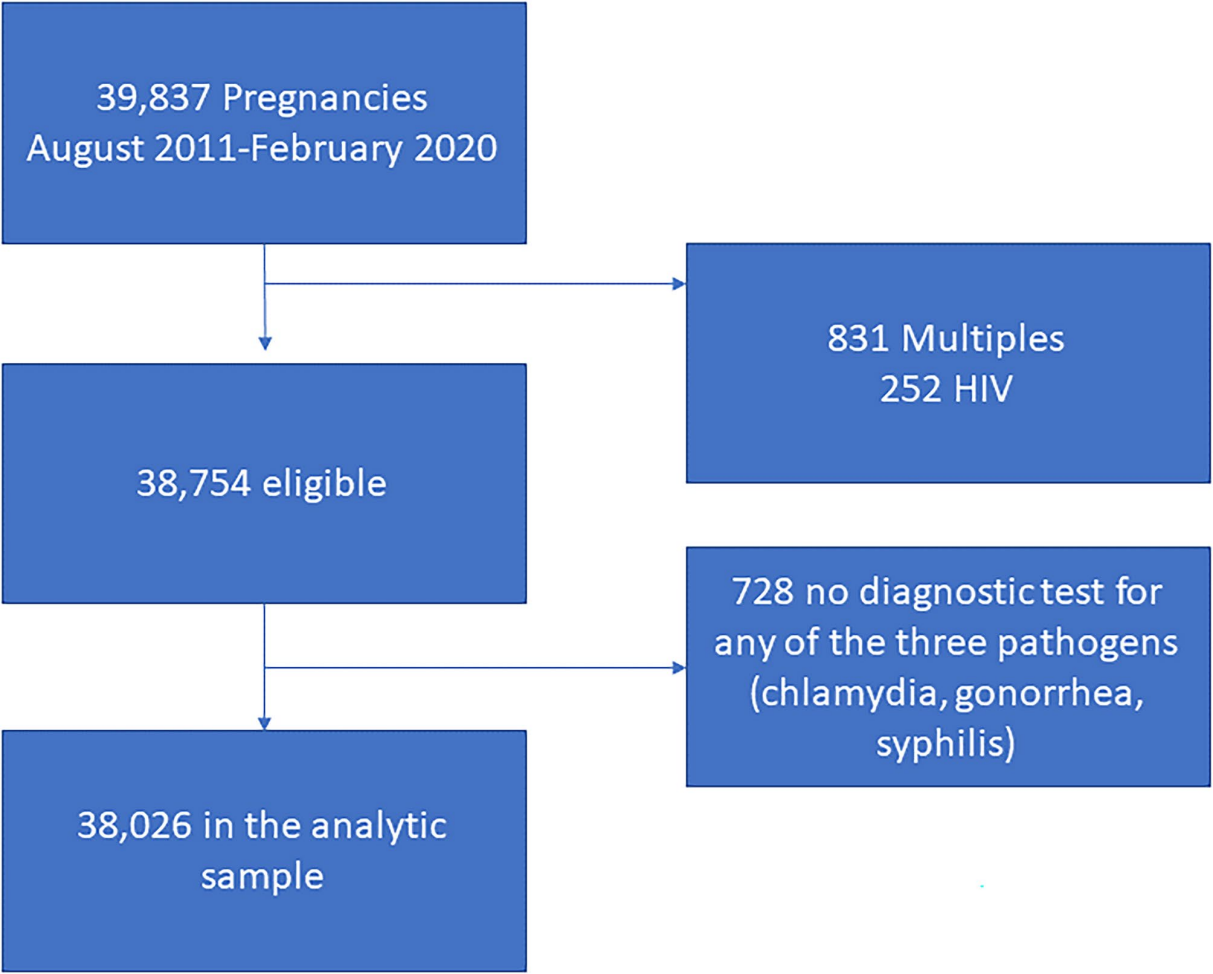
Flow diagram of sample size by exclusion criteria.

We identified 39,006 women with singleton and first recorded pregnancies delivering between August 2011 and February 2020 (Fig. 1). HIV is highly correlated with STIs, leading to multicollinearity in models and difficulty in distinguishing the effects of STIs vs. co-infection with HIV on pregnancy outcomes. A total of 252 women with HIV at the first or third trimester visit were excluded from analysis. This included any indication of HIV as we did not have information on viral load. We excluded 728 women who did not have a diagnostic test conducted for any of the three pathogens. After these exclusions, approximately, 91.1%, 90.6%, and 97.9% had a diagnostic test result for chlamydia, gonorrhea, and syphilis respectively. A total of 38,026 women were included in the final analysis.

The primary exposure of interest is a diagnosis with *Chlamydia trachomatis* and secondary exposures were a diagnosis of *Neisseria gonorrhoeae* and syphilis. Following standardized protocols, which tested all women at their first prenatal visit, vaginal swabs or urine are collected for *C. trachomatis* and *Neisseria gonorrhoeae* diagnosis using Nucleic acid amplification tests (NAAT)^31,32^. Standard guidelines were followed for treatment and re-testing in the third trimester. Syphilis was indicated in the database by rapid plasma regain (RPR), data on treponemal test was not available. We did not have information on the recommended 28 week retesting for high-risk individuals, thus, a new diagnosis at the first prenatal visit was included in the analysis. Data on other genital tract infections were available such as herpes simplex virus, hepatitis, bacterial vaginosis, and trichomonas. However, majority are only tested in symptomatic individuals. Data on routine testing for group B streptococcus was available.

The primary outcomes were HDP as defined by the American College of Obstetricians and Gynecologists^33^. Preeclampsia is defined by new onset of hypertension: “systolic blood pressure (BP) ≥ 140 mmHg and/or diastolic BP ≥ 90 mmHg on at least two occasions four hours apart after 20 weeks’ gestation but before labor and proteinuria (24 h urinary protein ≥ 300 mg or spot urine protein to creatinine ratio ≥ 30 mg/mmol creatinine or urine dipstick protein ≥  ++)^33^”. ACOG guidelines from 2013, state that “in the absence of proteinuria, the new onset of thrombocytopenia, renal insufficiency, impaired liver function, pulmonary edema or cerebral visual symptoms can be used for diagnosis.” Preeclampsia with severe features includes one or more of the following: (1) systolic blood pressure ≥ 160 mmHg; (2) diastolic blood pressure ≥ 110 mmHg; (3) proteinuria ≥ 5 g/24 h; (4) elevated liver enzymes or (5) platelet count ≤ 100,000^33^. Preeclampsia with delivery < 37 weeks gestation or term delivery was not examined as a separate outcome as both were previously examined in a smaller subset of this cohort^14^. We defined mild preeclampsia as women with preeclampsia as defined above, but without severe features. Gestational hypertension was defined as the new onset of elevated BP after 20 weeks gestation without proteinuria or severe features. Superimposed preeclampsia was diagnosed in women with underlying chronic hypertension who develop evidence of preeclampsia^33^.

Other variables of interest included self-reported sociodemographic variables: maternal age (< 25, ≥ 25), ethnicity (Hispanic, non-Hispanic) and race (Black or African American, White, American Indian/Alaska Native, Asian, Native Hawaiian or Pacific Islander, Other), education (high-school or greater, less than high-school, marital status (married, single), and insurance status (private, Medicaid/CHIP, no-insurance/other). Behavioral and health indicators including prenatal alcohol use (no, yes), drug use (marijuana, heroin, methamphetamine, or cocaine vs. no reported use) and tobacco use (no, yes), body mass index (BMI kg/m^2^ < 24.9, 25–29.9, 30+), mental health issues (no, yes—anxiety, depression, other disorders), chronic hypertension (no, yes), cardiovascular disease (no, yes), and diabetes (no, yes). Pregnancy history variables included prior pregnancies (nulliparous, parous), prior pregnancy complications (no, yes—history of spontaneous abortion, preterm birth), gestational age at 1st prenatal visit (≤ 12 weeks, 13–26 weeks, 27+ weeks), and infant sex (male, female).

### Statistical analyses

Associations between maternal characteristics and a diagnosis with chlamydia, gonorrhea, or syphilis was examined using log-binomial regression to calculate point prevalence ratios (PR) and 95% confidence intervals (CIs)^34^. We used directed acyclic graphs to theorize confounders^35^ including proxies for unmeasured confounders (e.g. race as a social determinant given well known associations with STIs and HDP) but excluding instrumental variables^36^. Model 1 adjusted for insurance type, maternal age (dichotomized as < 25 and ≥ 25 as this is the cut-point to determine high-risk for STIs), race/ethnicity (collapsed as Hispanic, White, Black, other), and education. A second model further adjusting for tobacco use, substance use, and co-infection (composite of other STIs, BV, or GBS) found similar effect estimates. For missing covariate data (range 0.01–9.4%), multiple imputation with 10 replications and fully conditional specification was utilized^37^. For the primary analysis, log-binomial regression was used to calculate relative risk (RR) and 95% CIs for the association between STIs and each HDP.

We conducted several sensitivity analyses. We examined whether associations were consistent among women with and without a documented CDC recommended treatment for each STI. We examined associations stratified by a STI diagnosis at the first prenatal visit only and among women who had a second diagnosis in the third trimester, which we refer to as persistent or recurrent infection. We examined chlamydia-gonorrhea co-infection, given mechanistic data suggesting that this combination exacerbates disease progression^10^. However, there were too few cases for analysis. We did not have data on specific gestational age of STI testing. However, per hospital protocols, women are tested at their first prenatal visit. As a proxy we assessed associations among women who received prenatal care in the first trimester and those who received care in the second and third trimesters. Lastly, to assess the effect of unmeasured confounding, E-values were calculated^38^. All analyses were conducted in SAS V 9.2, Cary, NC.

## Supplementary Information

Supplementary Tables.

## Data Availability

The data that support the findings of this study are available from Peribank but restrictions apply to the availability of these data, which were used under license for the current study, and so are not publicly available. To access data, interested parties should contact Peribank to request access to data.
