# Supplementary material for: Sexually transmitted infections and risk of hypertensive disorders of pregnancy
Source: Sci Rep. 2022 Aug 16;12:13904. doi: 10.1038/s41598-022-17989-0 (PMC9381495; doi:10.1038/s41598-022-17989-0)
Supplement: Supplementary file 1 — Supplementary Tables. [file 41598_2022_17989_MOESM1_ESM.docx]

| **sTable 1. E-values and confidence interval for the association between sexually transmitted infections (STIs) and hypertensive disorders of pregnancy (HDP)** | | | | | |
| --- | --- | --- | --- | --- | --- |
| Pathogen | Gest. Hypertension  **E-value (CI) | *Mild PE  **E-value (CI) | +PE with Severe features  **E-value (CI) | Superimposed PE  **E-value (CI) |  |
| Chlamydia  n = 1521 | 1.7 (1.4) | 1.4 (1.0) | 2.2 (1.4) | 2.6 (1.4) |  |
| Gonorrhea  n= 150 | 1.8 (1.0) | 1.4 (1.0) | 2.2 (1.0) | ++ |  |
| Syphilis  n = 268 | 2.4 (1.4) | 1.4 (1.0) | 1.0 (1.0) | 2.2 (1.0) |  |
| *Mild preeclampsia are individuals with preeclampsia who did not develop severe features  ** E-value estimate (Confidence Interval)  +Preeclampsia with severe features as defined by American College of Obstetricians and Gynecologists guidelines  ++ Sample size too small (n<5) | | | | | |

| **sTable 2. E-values and confidence intervals for the association between *Chlamydia trachomatis* and hypertensive disorders of pregnancy (HDP) by single diagnosis and persistent or recurrent infection.** | | | | |
| --- | --- | --- | --- | --- |
| Chlamydia | Gest. Hypertension  **E-value (CI) | *Mild PE  **E-value (CI) | +PE with Severe features  **E-value (CI) | Superimposed PE  **E-value (CI) |
| Single diagnosis  n= 1333 | 1.7 (1.0) | 1.0 (1.0) | 1.9 (1.0) | 2.8 (1.4) |
| Persistent infection  n= 192 | 1.7 (1.0) | 1.4 (1.0) | 3.4 (1.4) | ++ |
| *Mild preeclampsia are individuals with preeclampsia who did not develop severe features  ** E-value estimate (Confidence Interval)  +Preeclampsia with severe features as defined by American College of Obstetricians and Gynecologists guidelines  ++ Sample size too small (n<5) | | | | |

| sTable 3. Comparison of maternal characteristics between births in Harris County, TX 2010-2020 and Peribank (during study period 2011-2022) | | |
| --- | --- | --- |
| Variable | *Harris County, TX | **Peribank |
| Maternal race/ethnicity  Hispanic  NH-white  NH-Black  Asian  Other | 50.4%  22.5%  19.7%  6.9%  0.1% | 57.6%  21.4%  15.0%  4.8%  1.0% |
| Maternal age, years  <20  20-29  30-39  >=40 | 5.7%  47.8%  42.9%  3.5% | 5.9%  43.9%  45.7%  4.5% |
| Gestational age of first prenatal visit  First trimester  Second trimester  Third or no care | 59.9%  25.0%  15.1% | 61.6%  31.5%  6.85% |
| Preterm birth | ***11.6% | 9.8% |
| Total Cesarean births | 35.2% | 32.2% |
| *Data is from Harris County Texas, which includes Houston, TX, and surrounding areas. Source of the data is the March of Dimes, Births in Harris County Texas. <https://www.marchofdimes.org/peristats/data?top=2&lev=1&stop=10&reg=99&sreg=48&creg=48201&obj=3&slev=6>  **Peribank recruits from two hospitals within Baylor College of Medicine, a tertiary-care referral center, as well as the Texas Children’s Hospital Pavilion for Women, a private hospital, in Houston, TX.  ***Data was available for 2015-2020 | | |
